# Application of Nuclear Medicine Techniques in Musculoskeletal Infection: Current Trends and Future Prospects

**DOI:** 10.3390/jcm13041058

**Published:** 2024-02-13

**Authors:** Cristina Valero-Martínez, Valentina Castillo-Morales, Nieves Gómez-León, Isabel Hernández-Pérez, Esther F. Vicente-Rabaneda, Miren Uriarte, Santos Castañeda

**Affiliations:** 1 Rheumatology Service, Hospital Universitario de La Princesa, IIS-Princesa, 28006 Madrid, Spain; cristina.valmart@gmail.com (C.V.-M.); efvicenter@gmail.com (E.F.V.-R.); miren_uriarte@hotmail.com (M.U.); 2 Nuclear Medicine Service, Hospital Universitario de La Princesa, IIS-Princesa, 28006 Madrid, Spain; valentina.castillomo.hlpr@gmail.com (V.C.-M.); isabell.hernandez@salud.madrid.org (I.H.-P.); 3 Radiology Service, Hospital Universitario de La Princesa, IIS-Princesa, 28006 Madrid, Spain; nievesgleon@gmail.com; 4 Cathedra UAM-Roche, EPID-Future, Department of Medicine, Universidad Autónoma de Madrid (UAM), 28006 Madrid, Spain

**Keywords:** musculoskeletal infections, nuclear medicine, FDG-PET/TC, scintigraphy, osteomyelitis, spondylodiscitis, septic arthritis, periprosthetic infections

## Abstract

Nuclear medicine has become an indispensable discipline in the diagnosis and management of musculoskeletal infections. Radionuclide tests serve as a valuable diagnostic tool for patients suspected of having osteomyelitis, spondylodiscitis, or prosthetic joint infections. The choice of the most suitable imaging modality depends on various factors, including the affected area, potential extra osseous involvement, or the impact of previous bone/joint conditions. This review provides an update on the use of conventional radionuclide imaging tests and recent advancements in fusion imaging scans for the differential diagnosis of musculoskeletal infections. Furthermore, it examines the role of radionuclide scans in monitoring treatment responses and explores current trends in their application. We anticipate that this update will be of significant interest to internists, rheumatologists, radiologists, orthopedic surgeons, rehabilitation physicians, and other specialists involved in musculoskeletal pathology.

## 1. Introduction

Nuclear medicine (NM) studies play a pivotal role in the diagnosis and characterization of infections and inflammation in rheumatology. NM is a key tool for the identification and assessment of musculoskeletal (MSK) infections, including osteomyelitis (OM), infectious spondylodiscitis (SD), and prosthetic joint infections (PJI).

NM tests involve the intravenous administration of a radionuclide that emits radiation detectable by a gamma camera, enabling the evaluation of abnormal bone metabolism that manifests as areas of heightened radionuclide uptake [1,2,3]. These scans offer functional and metabolic insights during the initial stages of the disease, often preceding morphological changes. Selection of the appropriate study must be tailored to each patient and clinical scenario, as no single radionuclide agent is universally effective across all bone regions.

While conventional radionuclide imaging tests, such as the three-phase bone scan, radiolabeled leukocyte scan, and gallium scan, have historically been employed for diagnosing MSK infections, concerns about its diagnostic limitations have been raised [4]. To address this issue, fusion imaging scans, such as single-photon emission computed tomography combined with computed tomography (SPECT/CT) and positron emission tomography with CT (PET/CT), have been introduced. These techniques prove valuable in distinguishing between soft tissue and bone infections by providing morphological information [1,2,3,5]. This review offers a comprehensive examination of NM’s current and potential future applications for MSK infection management.

## 2. Fundamental Issues and Challenges Related to Conventional NM Techniques in Bone Infections

### 2.1. Bone Scintigraphy with Technetium-99m

Three-phase bone scintigraphy (BS) (perfusion, blood pool, and bone phases) with technetium-99 metastable (^99m^Tc) is the first diagnostic approach for MSK infections [1]. The examination is performed using ^99m^Tc complexed with a diphosphonate, either methylene diphosphonate (MDP) forming ^99m^Tc-MDP or hydroxydiphosphonate (HDP) forming ^99m^Tc-HDP. ^99m^Tc phosphonates localize in bone in proportion to osteoblastic activity, as seen at sites of bone remodeling, and uptake of the radiotracer occurs in many pathological processes (traumatized bone, malignancy, etc.), not only during infection, representing one of the main hurdles of the test. Uptake can also be found in other less common bone diseases, such as Paget’s disease, fibrous dysplasia, osteoid osteoma, or complex regional pain syndrome [1,2,3,4]. One of the advantages of this test is that the images are obtained 2–4 h after radiotracer injection, making it an accessible and rapid test [3,4].

Three-phase BS with ^99m^Tc is often used to rule out peripheral OM in bones unaffected by underlying conditions and is useful for detecting missed multifocal joint infections that have gone unnoticed. OM is diagnosed when focal hyperperfusion and increased bone uptake are found in the area of interest in delayed images (in all three phases), which allows its distinction from cellulitis (high in the first two phases) or septic arthritis (hyperemia in the synovial vessels) [1,3,5,6]. The interest of this test lies in its high negative predictive value of OM and high sensitivity (73–100%), despite its poor specificity [1,3,7]. In situations with manipulated bone (fracture, trauma, surgery, malignancy, inflammatory arthritis, etc.), sensitivity remains high (89–100%), but specificity is usually low (around 10%) [8]. In patients with PJI, sensitivity is lower (around 50%) though specificity increases (around 70%) [9,10,11,12]. An important issue is that the test can be falsely positive, especially in the first few years after prosthesis replacement, because osteoblastic activity is increased. Positive bone scans should be interpreted with caution within two years of hip and shoulder replacement surgery and within five years of knee replacement [2,8,13].

In the spine, three-phase scans are also difficult to interpret due to planar imaging, which prevents visualization of overlying and vascular structures [1,2,3,4,5,6]. Therefore, the test is unable to discriminate soft-tissue infections that often accompany SD and sometimes mimic this condition [14]. Three-phase BS with ^99m^Tc has high sensitivity (90%), but moderate specificity (36%) in detecting SD [15]. The test has other limitations as it can sometimes show decreased rather than increased uptake in SD. The causes are believed to be inadequate blood supply (induced by pus or vasospasm), destructive bone lesions, or non-pyogenic infection. Furthermore, false-negative results have been reported in elderly patients with SD, possibly because of regional ischemia secondary to arteriosclerotic disease [16]. Another problem is that uptake may remain abnormal even after the infection has resolved due to ongoing bony remodeling during healing, as part of the normal reparative process [2,17,18].

### 2.2. Bone Scintigraphy with Gallium Citrate (Ga-67)

Gallium citrate Ga-67 BS (^67^Ga-BS) has been classically used for years to localize bone infections and is usually used in combination with three-phase BS. ^67^Ga-BS is almost exclusively limited to the spine and is still frequently used to rule out SD in clinical practice [6]. ^67^Ga-BS is more specific in diagnosing SD than three-phase BS, and one of its strengths is that it can detect soft tissue infections with a reported sensitivity and specificity of 61% and 73%, respectively [19]. Moreover, its combination with three-phase BS increases the sensitivity, especially in the elderly [15,20]. In peripheral OM or PJI, it is a disused test because of its low sensitivity [21]. Nevertheless, three-phase BS combined with ^67^Ga-BS seems to provide greater specificity than the triphasic study alone, although it is not routinely recommended for the diagnosis of peripheral OM or PJI [6,9,22,23].

One of the main limitations of this test is that the procedure requires two different tracers, as well as multiple imaging sessions, sometimes extending over 48–72 h after injection, which causes delay in diagnosis and treatment [1,2,6]. Other problems of the test include relatively low image quality and false positives in various conditions, such as trauma or tumors [1].

### 2.3. In Vitro and In Vivo Labeled Leukocyte Bone Scintigraphy

Radiolabeling of leukocytes has been successfully used to localize MSK infection. In most situations, the labeled cells are neutrophils, which explains why labeled leukocyte BS is more sensitive for identifying neutrophil-mediated inflammatory processes, such as bacterial infections [1,2,3,4], and less beneficial for parasitic, viral, or mycobacterial infections [24,25]. Indium-111 (^111^In) and ^99m^Tc-labeled hexamethylpropylene amine oxime (99mTc-HMPAO) are the most commonly used radiotracers. Despite the several differences between these two isotopes, no statistically significant differences in their accuracy have been found [7,26]. ^111^In shows label stability with normal limited distribution and the possibility of performing delayed imaging; however, the resolution of images is low and requires 16–30 h between injection and imaging. In contrast, ^99m^Tc-HMPAO has a large normal distribution but offers high-resolution images and low radiation. Disadvantages include label instability and a short half-life of ^99m^Tc, which limits delayed imaging [1,2,3,4,24].

As with three-phase BS or ^67^Ga BS, increased uptake may also occur with this technique in other non-infectious conditions (for example, after fracture or joint arthroplasty), therefore, decreasing specificity. To maximize the specificity of the test in cases with variability in bone marrow distribution, such as prosthetic joints, complementary imaging of the bone marrow with ^99m^Tc sulfur colloid is typically performed. The study is considered positive for infection when activity is only present in the leukocyte image and not in the marrow image, with an overall accuracy of over 90% [24,27]. Combining labeled leucocyte BS with bone marrow BS has been reported to increase the detection of PJI and reduce false-positive cases, with accuracy ranging from 83-98% for both knee and hip PJI [27,28,29,30].

Labeled leukocyte BS is considered the first test of choice or the gold standard in patients with suspected OM who present with manipulated bone or prosthetic joints because of its high sensitivity and specificity [25,31]. This scan is frequently used in combination with three-phase BS and can improve the specificity of conventional three-phase BS by up to 90%. Labeled leukocyte BS is useful in the differential diagnosis of PJI and reactive changes and/or aseptic loosening [23]. It has been proposed to proceed directly to this scan within the first 2 years of index surgery [13,25,31]. Labeled leukocyte BS has disadvantages including delayed results and in vitro handling and may not be viable in leukopenic patients [1].

In contrast to other sites of the skeleton, labeled leukocyte BS is not useful for spinal OM because of its low sensitivity and specificity, and should not be used for the diagnosis of SD because the occlusion of the microcirculation of the affected bone may enhance the uptake of the healthy adjacent bone marrow [6]. This can result in a pattern of decreased or absent activity, which cannot be differentiated from other causes of marrow replacement [1,2,32]. Additionally, planar images are not always able to discriminate between bone and soft tissue infections because they frequently occur in SD [25].

In vivo labeled leukocyte BS is performed using ^99m^Tc-labeled monoclonal murine anti-granulocyte antibodies against surface antigen-95 (besilesomab) or antigen-90 (sulesomab) to trace granulocyte precursors and leukocytes, respectively. In vivo labeling of leukocytes with antibodies requires only a single injection, whereas in vitro labeling of isolated cells is a time-consuming, multistep process. In vitro and in vivo labeled leukocyte scans have similar accuracies [1,2,3,4]. Several studies have shown that in vivo labeled leukocyte agents, when used in conjunction with conventional three-phase BS, significantly improve specificity and accuracy in the diagnosis of OM in traumatized or prosthetic bone. Additionally, the negative predictive value of this approach is very high (92–100%) [33,34,35,36,37,38,39,40]. Although this technique has been proven to be precise, effective, and safe, its sensitivity is limited by its high physiological uptake in the bone marrow. Additional disadvantages of the technique include the possibility of allergic reactions and that it is not useful for monitoring the treatment since it cannot be repeated. Therefore, it has not yet gained widespread use in vivo [41].

## 3. The Introduction of Hybrid NM Techniques in Bone Infections

### 3.1. SPECT/CT plus ^99m^Tc, ^67^Ga or Labeled Leucocyte Scintigraphy

**Figure 1:**
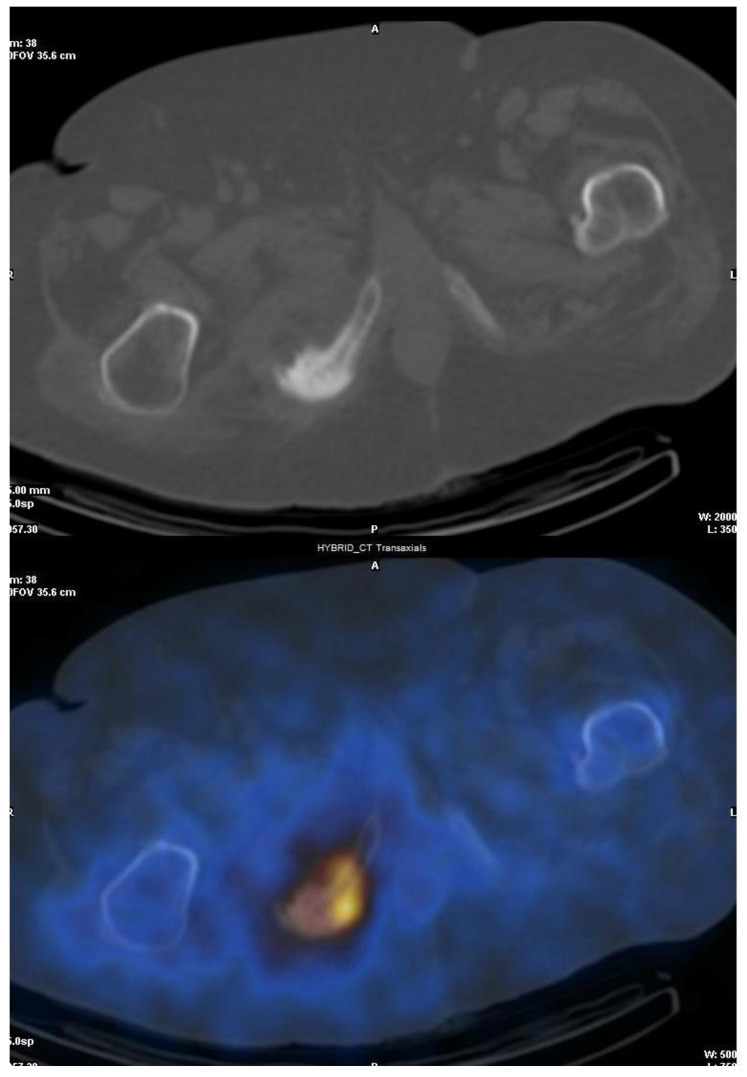
Osteomyelitis of the ischiopubic ramus by *Morganella morganii* and *Proteus mirabilis*. A 73-year-old male patient with a previous diagnosis of a polymicrobial stage 4 bedsore over ischial tuberosity who developed an osteomyelitis of the ischiopubic ramus and secondary bacteremia by *Morganella morganii* and *Proteus mirabilis*. A SPECT-CT with ^67^Ga was performed showing pathological gallium deposits on the right ischiopubic ramus.

**Figure 2:**
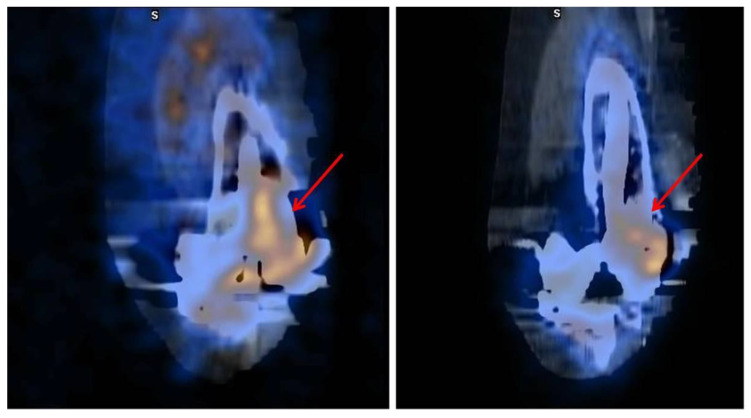
Prosthetic left knee infection detected by scintigraphy (^99m^Tc-HDP and ^67^Ga) and SPECT-CT. Combination of scintigraphy with ^99m^Tc-HDP and ^67^Ga with SPECT-CT in an 84-year-old male patient with prosthetic left knee infection and multiple comorbidities. The scan showed the focus of increased bone uptake in the external femoral condyle, with extensive associated gallium deposition, compatible with an infectious process (red arrows).

The use of SPECT/CT significantly improves the accuracy of conventional scintigraphy and labeled leucocyte scans and allows anatomical localization of bone infection, leading to a better differentiation between bone involvement and surrounding soft tissue infection (Figure 1 and Figure 2) [21,31,42,43,44]. This imaging test localizes the radiopharmaceutical uptake seen on planar images precisely, improving disease detection and changing OM diagnosis in around 60% of the patients [45,46,47,48,49,50,51].

**Figure 3:**
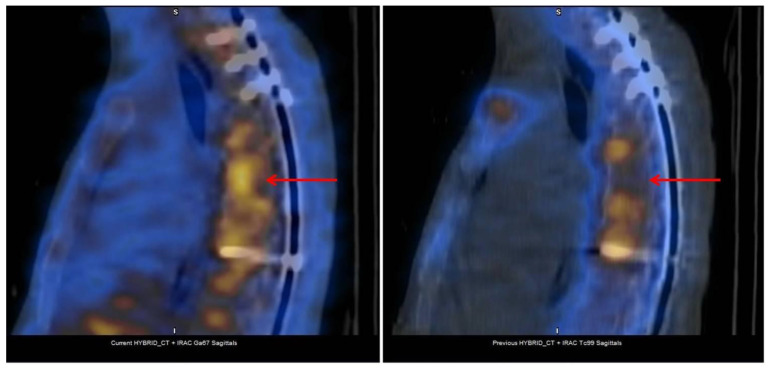
Spondylodiscitis by *Brucella* at T7. A 44-year-old man with dorsal spondylodiscitis by *Brucella*. The patient was previously diagnosed with polyarteritis nodosa and treated with mycophenolate mofetil and glucocorticoids. Combination of scintigraphy with ^99m^Tc-HDP and ^67^Ga with SPECT-CT shows pathological gallium deposits at the level of T7 (red arrows) (axial view) compatible with infectious activity and inflammatory/degenerative bone remodeling changes in the rest of the dorsal vertebral bodies.

The use of SPECT/CT has been standardized in clinical practice to confirm the findings of conventional scintigraphy and to provide more information about the infection, so it is recommended whenever the planar images are positive [22]. In SD, SPECT-CT scintigraphy with ^99m^Tc and ^67^Ga tracers increased the sensitivity to 92% compared to scintigraphy alone, and a high accuracy (97%) of combined bone/^67^Ga SPECT/CT was shown (Figure 3) [52]. Love et al. [15] stated that abnormal uptake in two contiguous vertebrae on SPECT images was the most accurate criterion (71%) for detecting SD. In PJI, the joint reliability of SPECT-CT with conventional scintigraphy is between 83–98%, although some studies have found low sensitivities, probably related to a bias in the frequency in favor of subacute or chronic infections [1]. A study investigating SPECT-CT imaging with ^99m^Tc-besilesomab showed 66% sensitivity, 60% specificity, and 61% accuracy. When planar images were interpreted together with SPECT-CT, the sensitivity remained unchanged, although the specificity and accuracy increased to 73% and 77%, respectively [53].

### 3.2. Fluorine-18-Fluorodeoxyglucose (18F-FDG) with Positron Emission Tomography (PET/CT)

Positron emission tomography (PET) is an NM imaging modality that uses 18F-FDG (or FDG) as a tracer with high-resolution tomographic images. FDG is a glucose analog transported into cells via glucose transporters and is phosphorylated by hexokinase to FDG-6 phosphate. Greater 18F-FDG uptake is seen in areas of inflammatory cell infiltrates (neutrophils or macrophages in the acute and chronic phases, respectively), indicating increased glucose use by these cells at the site and time of infection. 18F-FDG is a small molecule that rapidly enters poorly perfused regions, and the procedure is complete within 1–2 h [54,55,56]. 18F-FDG PET allows for the development of high-resolution images that localize 18F-FDG accumulation and low radiation loss. 18FDG uptake in spinal infections is higher than in the normal bone marrow. 18F-FDG PET combined with CT has been used for the diagnosis and follow-up of OM and SD showing promising results, being superior to other imaging modalities [56].

**Figure 4:**
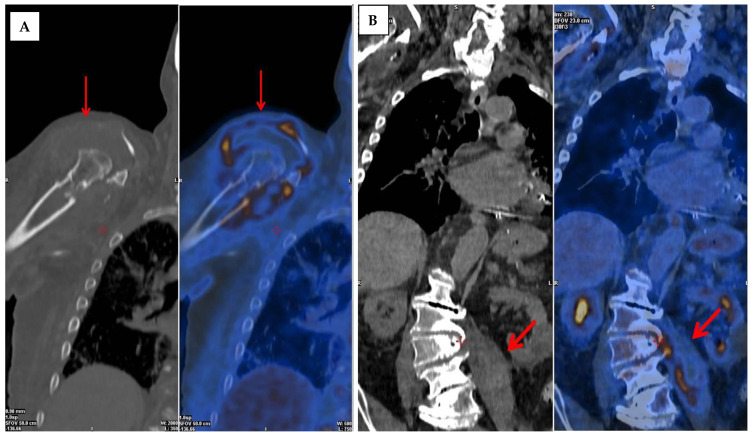
Spondylodiscitis at L2–L4 level and septic arthritis of both shoulders secondary to infective endocarditis due to methicillin-resistant *Staphylococcus aureus*. 18F-FDG PET/CT in 85-year-old male patient with lumbar spondylodiscitis (L2–L4) and septic arthritis of both shoulders, with bacteremia secondary to infective endocarditis on prosthetic aortic valve due to methicillin-resistant *Staphylococcus aureus*. The scan showed a significant FDG uptake in both glenohumeral joints (**A**) and at the level of the left psoas (**B**) in relation to a significant abscess with associated L2–L3 spondylodiscitis. Furthermore, elevated focal uptake was found in the aortic valve, compatible with suspected endocarditis (red arrows).

18F-FDG PET/CT has been proposed as a useful tool to provide more accurate diagnostic information in non-traumatized peripheral OM and can also help in the discrimination between soft tissue and bone infection by providing morphological information (Figure 4) [22]. Wang et al. performed a meta-analysis on 18F-FDG PET/CT as a diagnostic test for peripheral OM, which showed a higher pooled sensitivity and specificity compared with three-phase ^99m^Tc BS and “in vitro and in vivo” labeled leucocyte scan [26]. Nevertheless, there is insufficient evidence on PET/CT for acute OM, and most of the available studies are focused on chronic OM, where 18F-FDG PET/CT has the highest sensitivity and specificity compared to other scans, due to the accumulation of FDG in activated macrophages (the predominant cell type found in chronic infection) [17,21,57,58]. One of the challenges of this type of scan is that it can be difficult to distinguish between active infection, inflammation, and malignancies [59]. However, an intense and prolonged focal uptake of FDG in the bone marrow should always raise suspicions of malignancy [59].

The role of 18F-FDG PET/CT in the diagnosis of spinal OM has been widely evaluated with satisfactory results (89–97% sensitivity and 88% specificity) [60] as an adjunct to MRI [20,32,43,61,62,63,64,65,66], and the good diagnostic accuracy of the test has been demonstrated (70–100%) [67,68]. A meta-analysis using PET/CT in SD showed a pooled sensitivity of 94.8% and specificity of 91.4% [23]. The advantages of 18F-FDG PET/CT include its superior spatial resolution, better characterization of the extent of infection, differentiation of severe spinal degenerative changes from infection, and better detection of other sites of embolic infection [64,66,69,70]. The differentiation between infectious and degenerative changes can pose a challenge, typically requiring a combination of precise localization of FDG uptake (e.g., intervertebral disc in the context of SD) along with comprehensive clinical investigation and physician’s expertise [59]. In the spine, 18F-FDG PET/CT is useful for differentiating acute osteoporotic fractures (without FDG uptake) from pathological fractures due to malignancies or inflammatory diseases (with FDG uptake). The literature suggests that 18F-FDG PET/CT may be superior to ^67^Ga and ^99m^Tc scans in diagnosing SD and paraspinal soft tissue infection and in differentiating infection from advanced arthritic changes [20,32,43,61]. Other studies also showed better accuracy for SD with 18F-FDG PET/CT compared to SPECT/CT with ^99m^Tc, ^67^Ga [48], or 99mTc-besilesomab scans [71].

An important clinical application of 18-FDG PET/CT is to investigate suspected infection in traumatized bones or osteosynthesis material. Although increased FDG uptake may also occur during the healing of fractures, distinguishing between fracture healing and infection is generally based on the location of the elevated FDG uptake. Bone regeneration typically induces increased FDG uptake across the complete surface of the fracture line, whereas infection manifests as a focal elevation of FDG uptake [72]. One of the limitations of 18F-FDG-PET/CT is its low specificity for post-surgical changes or metallic implants [62]. Compared with labeled leucocyte scan, 18F-FDG PET/CT provides the same sensitivity, but lower specificity for diagnosing PJI in some studies [10,73,74,75,76], probably due to the subacute or chronic nature of most PJI, generally involving monocytes and lymphocytes. Published studies show less specificity and accuracy for knee than for hip prosthetic infection, since post-surgical uptake in the knee may be chronic in asymptomatic patients [73,77,78,79]. There are controverted results on the ability of 18F-FDG PET/CT to differentiate infection from aseptic loosening or inflamed prosthesis, with a high rate of false-positive results [50]. The consensus statement concluded that based on the available literature, 18F-FDG PET/CT has a high negative predictive value and may effectively rule out PJI [13], but if positive, it may not be confirmatory of PJI [80], so it should be interpreted with caution.

### 3.3. Fluorine-18-Fluorodeoxyglucose (18F-FDG) PET/MRI

Whole-body 18F-FDG-PET/MRI is an emerging hybrid imaging technique that offers the opportunity to combine in one examination the most sensitive information on morphology, bone marrow cellularity and vascularization, and metabolic activity; however, the real upgrade in patient care, given the cost and the need for double expertise, is still under investigation. Given that MRI does not emit ionizing radiation, substituting CT with MRI has the potential to curtail radiation exposure for patients, providing higher soft tissue contrast, such as in cartilage and muscle. 18F-FDG PET/MRI has been extensively explored in various diseases, including cardiac sarcoidosis, myocarditis, endocarditis, inflammatory bowel disease, transplant-related complications, and tuberculosis. Recent studies have particularly focused on application in cardiovascular conditions, with a primary focus on atherosclerosis. The review by Kirienko et al. [81] noted a rising trend in publications related to bone and joint diseases from 2018 to 2022. This review confirmed the potential of PET/MRI to have a positive impact on the management of infectious diseases and inflammatory conditions.

In MSK infections, Hulsen et al. evaluated 18F-FDG PET/MRI scans for suspected OM and reported a sensitivity, specificity, and accuracy of 78%, 100%, and 86%, respectively, similar to those reported in the literature with 18F-FDG PET/CT [82]. In SD detection, 18F-FDG-PET/MRI improves the sensitivity and specificity (100% and 88%, respectively) compared to assessment using MRI alone, especially in patients with inconclusive clinical or MRI findings [83]. 18F-FDG PET/MRI has also been investigated with successful results in assessing treatment response in SD and has demonstrated a significantly higher 18F-FDG uptake in non-cured SD [84]. In fact, Jeon et al. [84] showed that patients who were not cured exhibited a significantly higher intensity on T2-weighted fat saturation MRI images in both bone and soft tissue compared to the reference. The same group identified possible predictive markers of intervertebral auto-fusion using 18F-FDG PET/MRI after successful antimicrobial treatment in SD. Patients with auto-fusion exhibited higher erythrocyte sedimentation rate and 18F-FDG uptake at six months and extensive edematous changes on T2-weighted fat saturation MRI [85].

In PJI, a study conducted by Henkelmann et al. [86], evaluated 18F-FDG PET/MRI in 10 patients with suspected PJI of the hip and knee compared with surgical or biopsy specimens. 18F-FDG-PET/MR accurately diagnosed all cases with a sensitivity and specificity of 100%, demonstrating superior performance in soft tissue evaluation compared to periprosthetic bone margin or bone marrow assessments.

### 3.4. Fluorine-18-FDG-Labeled White Blood Cells (18F-FDG-WBC) PET/CT

Leukocytes have also been labeled with F-18-FDG in combination with PET/CT, offering the potential to enhance both sensitivity and specificity without causing toxicity or adverse effects. Another potential improvement with this technique is its capacity to enhance the differentiation between aseptic and septic inflammation, addressing a limitation not adequately covered by 18-FDG PET/CT. In this procedure, red blood cells are separated through sedimentation, and the WBC-rich plasma is centrifuged to obtain a pellet with WBCs. This pellet is then reconstituted in heparinized normal saline and labeled with 18F-FDG at 37 °C for white blood cell imaging. Approximately 148–185 MBq of 18F-FDG-labeled WBCs are administered to the patient, with the optimal imaging time being 120 min post-injection. However, there are some limitations to consider, including the variable efficiency of FDG, which is lower than that of In-111-oxine, the short half-life of only 110 min for F-18, the increase in the total duration of the investigation, off-site labeling requirements, and the absence of delayed imaging [54,87]. The clinical indications investigated with 18F-FDG-WBC to date include infections in renal cysts, in peripancreatic fluid collections in cases of pancreatitis, as well as MSK infections [87].

A meta-analysis with 18F-FDG-WBC PET or PET/CT has been carried out to evaluate suspicious multiple infectious diseases [88]. A higher diagnostic performance was found using 18F-FDG-WBC PET compared to conventional imaging methods (CT or MRI) and 18F-FDG PET/CT. The pooled (per-patient based) sensitivity and specificity for localizing infective foci in various organs were 86.3% and 92%, respectively. This meta-analysis did not identify a specific standardized uptake value (SUV) cut-off that could reliably indicate the presence of infection.

In MSK infections there are some evidence in the literature on the use of 18F-FDG-WBC PET in PJI or OM [87]. Manda et al. [89] studied the value of 18F-FDG-WBC PET/CT compared with conventional 18F-FDG PET/CT in 33 patients with fever of unknown origin or suspected PJI, and all patients underwent both scans. They demonstrated significantly higher diagnostic accuracy with 18 F-FDG WBC PET/CT in comparison to 18 F-FDG PET/CT for the detection of infection. Aksoy et al. [90] assessed 46 patients with painful joint arthroplasty using 18F-FDG-WBC PET/CT, demonstrating a high diagnostic performance with a sensitivity of 93.3% (14/15), specificity of 97.4% (38/39), and positive and negative predictive values of 93.3% and 97.4%, respectively. Rastogi et al. [91] evaluated the efficacy of 18F-FDG-WBC PET/CT in 23 patients with Charcot’s neuroarthropathy and suspected diabetic foot OM. They reported a sensitivity and specificity of 83.3% and 100%, respectively, for 18F-FDG-WBC PET/CT, as opposed to 83.3% and 63.6 for contrast-enhanced MRI.

## 4. Which Is the Most Appropriate Test for the Diagnosis of Bone Infections: Imaging, NM Scans, or Both?

Bone infections are considered one of the few rheumatology emergencies. The diagnosis of MSK infections remains a challenge for clinicians, and early identification of the infection is crucial to establish antibiotic treatment as soon as possible, because its delay can have devastating consequences for the bone, such as osteonecrosis and bony destruction.

In suspected acute MSK infections, such as septic arthritis or PJI, the process is usually easily identified based on clinical examination, radiography, laboratory data, or microbiological samples (synovial fluid, blood cultures, bone biopsy, etc.) that identify the causative infectious agent [18]. However, other imaging techniques may have added value for diagnosis by providing complementary information or facilitating interventional procedures. For example, ultrasonography is also helpful to detect fluid (joint effusions, abscesses, tenosynovitis) and guide arthrocentesis, but lacks value in OM due to its inability to penetrate the cortex of the bone [3,92,93], whereas MRI is used to rule out OM and evaluate the extent of MSK infection, and CT is useful to diagnose infections of the fibrocartilaginous joints (pubic symphysis, sacroiliac, or sternoclavicular joint) [94,95]. Radionuclide tests, which are highly time-consuming, are normally avoided in the initial diagnostic workup of septic arthritis unless there is suspicion of an underlying disease or when previously mentioned techniques do not show conclusive results. In later phases of bone infections, such as subacute or chronic processes (more than 6–8 weeks), differentiating the conditions is challenging [92]. In these scenarios, imaging tests play an essential role in confirming and localizing the disease. Spinal infections are often limited to the vertebral body and intervertebral disc, but can also involve the epidural space, posterior elements, and paraspinal soft tissues with abscess formation [15,17,32]. For this reason, imaging techniques are required to identify the structures adjacent to the bone.

When there is a suspicion of MSK infections, the first imaging that should be carried out is radiography, because it is readily available, inexpensive, and provides a comprehensive examination of a specific anatomical area. Radiography can also rule out the possibility of fractures and tumors as the underlying causes of swelling or pain [3,22,92,93]. Radiographs lack sensitivity and specificity for the evaluation of bone infections, but some signs, such as the presence of articular or soft tissue gas with joint effusion or soft tissue edema, are indicative of infection (septic arthritis or necrotizing fasciitis). Common radiographic findings in acute OM include erosion and periosteal reactions, whereas bone sclerosis is commonly associated with chronic OM. In PJI, radiographs provide information related to the extent of fracture healing, including the identification of delayed union, heterotopic ossifications, and assessment of joint alignment and surgical hardware integrity [3,93,96].

For suspected unifocal cases of OM and SD, the preferred imaging modality or gold standard test is MRI, based on its excellent anatomical details and high contrast resolution to determine the extent of bone involvement. Additionally, MRI has direct multiplanar imaging capability and high sensitivity and specificity for detecting early infections or bone destruction without exposing the patient to ionizing radiation. MRI is also feasible with metallic implants “in situ” [3,5,20,32,49] and provides an excellent evaluation of the adjacent soft tissues. It can identify muscular involvement, abscesses, fistulas, and vascular complications, and can differentiate other conditions including bone tumors [22,93]. In axial assessment, MRI allows for the possibility of performing the evaluation of the bone marrow with multiplanar sequences and the visualization of neural structures and soft tissue, including epidural abscesses [97]. One problem is that surgical hardware has the potential to generate substantial susceptibility artifacts, which can lead to reduction in and/or distortion of the visual representation of adjacent osseous and soft tissue structures [96]. Other limitations include its high cost and the lengthy acquisition time, which typically ranges from 20 to 90 min. Moreover, false positives can occur due to edema, and bone marrow signal abnormalities may persist for months following injury or surgery, which can potentially distort the results [3,8].

CT imaging can be used to guide a biopsy or procedural planning in suspected OM or SD when MRI is not available. In chronic OM, CT is most useful for characterizing osseous changes, such as periosteal reaction, destructive bone changes, sequestrations, and fistulous tracts, due to its low sensitivity. CT may also help differentiate cellulitis, myositis, tenosynovitis, abscess, and septic arthritis, and is more effective than MRI for identifying sequestrations, foreign bodies, and soft tissue/foci of intraosseous gas [3,22,92,93,98]. In patients with hardware, CT is a good imaging modality for the evaluation of fracture non-union or osteolysis adjacent to the implanted hardware [93,96]. Following the onset of OM symptoms, noticeable bone destruction may take 3–6 weeks to develop, potentially causing diagnostic delay. CT imaging has a high false-negative rate for epidural abscesses and a poor definition of early structural changes, making it an uncommon diagnostic tool in this scenario. Further drawbacks associated with CT include its ionizing radiation and the potential for image degradation owing to metallic artifacts [3,93,94,95,96,98].

Radionuclide imaging is not typically the first-line diagnostic approach for MSK infections. It is usually utilized to complement the results of imaging tests or in situations where imaging tests are contraindicated or inconclusive. Consequently, certain tests, such as bone scintigraphy, have been replaced by others, including MRI and imaging tests that use radionuclides, such as 18F-FDG PET/CT. The usefulness of the radionuclide test in cases of suspected OM or SD is controversial because of its low specificity [2,3]. Therefore, physicians typically request imaging tests and overlook radionuclide tests in clinical practice. It should be noted that SPECT and planar scintigraphy are more accessible and cheaper than other imaging tests and could still play an important role in the diagnostic process of bone infections in certain cases.

If there is a low probability of OM, using three-phase ^99m^Tc BS with SPECT-CT can be helpful to quickly rule out OM thanks to its high negative predictive value, making it unnecessary to wait for an MRI, which may not be easily available at all centers. This can prevent delayed diagnosis due to the unavailability of MRI. The radionuclide test can also be helpful in suspected OM when there is suspicion of multifocal disease and MRI is contraindicated, or when the infection is associated with orthopedic hardware or chronic bone abnormalities from trauma or surgery [3,5,22]. In cases of suspected late PJI or OM in the manipulated bone (e.g., trauma or recent surgery), three-phase ^99m^Tc BS has low specificity owing to osseous remodeling and persistent uptake. In these cases, radionuclide tests including three-phase ^99m^Tc BS in combination with In ^111^-labeled leukocytes with or without ^99m^Tc sulfur colloid in combination with SPECT/CT are frequently used with high accuracy [92].

The differential diagnosis between infection in orthopedic hardware and aseptic loosening is essential because the treatment of these two complications is very different, and radionuclide testing may help determine a definitive diagnosis [3,9]. In cases of ambiguous results for septic loosening or OM, the inclusion of a sulfur colloid scan can provide valuable insights into leukocyte accumulation near orthopedic implants [23]. 18F-FDG PET/CT has also emerged as a promising imaging modality and alternative to MRI in diagnosing active OM, with comparable results in terms of accuracy [21,22,57]. However, as with MRI, it is an expensive and even less accessible technique, which makes its use difficult in cases where the time until diagnosis can be decisive. Additionally, in hybrid techniques, artifacts stemming from metallic implants restrict visibility in CT scans, and such artifacts may contraindicate the use of MRI [92,93].

In SD, CT and MRI may be difficult to interpret and are also limited by their low specificity and false-positive results in several situations: severe degenerative disease, insufficiency fractures, pseudoarthrosis, inflammatory processes, vertebral amyloidosis, neuropathic arthropathy, and erosive intervertebral osteochondritis [97]. Whenever MRI is contraindicated or their results are inconclusive, radionuclide imaging may overcome these problems [15,20]. Combining three-phase 99mTc BS with ^67^Ga BS is an alternative standard of care to MRI [6,20,52], and published studies have demonstrated that their combination is equivalent to MRI [52,99,100]. Several studies have found that 18F-FDG-PET/CT has higher sensitivity and specificity than MRI for detecting SD [63,64,101,102,103] and is capable of discriminating degenerative changes (Modic changes) from disk space infection, differentiating it from MRI [59,60]. This technique has a higher accuracy than MRI when performed two weeks after symptom onset [63,64]. Other studies concluded that MRI was the modality of choice for diagnosing epidural and spinal abscesses, while 18F-FDG-PET/CT had higher sensitivity in diagnosing paravertebral and psoas abscesses and embolic infectious disease [63,64]. Gratz et al. [61] showed that 18F-FDG-PET/CT is superior to MRI in post-operative infection cases in the presence of post-operative hardware because functional images are not affected by metal artifacts. Recent consensus statements recommend 18-F-FDG PET/CT as the radionuclide test of choice in suspected SD, especially after inconclusive or misleading MRI [6,55,93,104], or in patients with a history of surgery and metallic implants in whom MRI is contraindicated [105].

**Figure 5:**
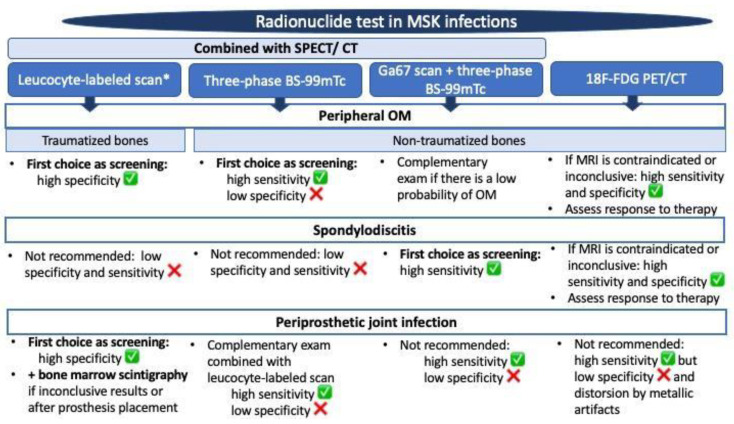
Summary of the more appropriate radionuclide tests for diagnosis of osteoarticular infections. * Leucocytes are usually labeled with 111Indium oxyquinolone (111In) or 99mTc-exametazime (99mTc). Abbreviations: BS: bone scintigraphy; CT: computerized tomography; 18-F-FDG: fluorine-18 fFluorodeoxyglucose; MRI: magnetic resonance imaging; OM: osteomyelitis; PET: positron emission tomography; SPECT: single-photon emission computerized tomography.

Figure 5 shows a summary of the more appropriate radionuclide examinations for the diagnosis of MSK infections according to the literature-based evidence.

## 5. Monitoring Musculoskeletal Infections: The Role of NM

Assessing the response to antibiotic therapy in MSK infections is often challenging, and it takes a long time to determine if there is complete healing of the disease, even months after the completion of therapy. One of the main problems in these cases is that inflammatory markers are not valid enough to predict the therapeutic response, and standard imaging has low sensitivity. MRI is not usually indicated for the follow-up of bone infections, as the results can be misleading [105].

NM scans have not been classically used for the follow-up of MSK infections because of the low specificity of scintigraphy for assessing therapeutic responses in the early phases. Uptake in peripheral and axial OM can be positive even months after the bone infection has resolved due to persistent bone remodeling, especially in patients with recent surgery or prosthesis placement [2,8,13,17,18].

Researchers have explored the utility of 18-FDG PET/CT examinations to address these limitations for monitoring antibiotic therapy in bone infections. A study conducted in children with acute OM found that 18-FDG-PET/CT was effective in distinguishing between the infection and the reparative processes in the MSK system after antibiotic treatment [106]. Additionally, PET/CT with 18-FDG appears to be effective in monitoring early responses to treatment in patients with SD [32,107,108]. This modality has a sensitivity of 85.7% and a specificity of 82.6% according to a study, surpassing those of MRI [109]. Riccio et al. [107] concluded that after antibiotic therapy, 18F-FDG uptake in bone or soft tissue indicates active infection. They also suggested that 18F-FDG uptake limited to the margins of a destroyed disc should not indicate a persistent infection. Normalization of SUVmax is a powerful predictor of residual disease and response to antibiotic therapy with higher sensitivity in some studies [108,109,110]. Russo et al. described the UDIPROVE protocol in the management of SD and showed that the combined utilization of 18F-FDG PET/CT and C-reactive protein (CRP) exhibits heightened precision in tracking therapy response, thus facilitating the timely assessment of treatment efficacy [111]. Righi et al. conducted an FDG-PET/CT scan on SD patients after at least 2 weeks of antibiotic treatment. Clinical improvement was associated with a significant reduction in all FDG-PET/CT parameters, with higher sensitivity and specificity than MRI [112]. Jeon et al. showed an earlier recovery pattern with FDG-PET/MRI than with MRI [84]. Consensus documents recommend a follow-up 18F-FDG PET/CT scan to evaluate the response to antibiotic therapy in SD or suspected recurrence [55,104].

## 6. Potential Developments for NM in Bone Infections: New Radiopharmaceuticals or Techniques

With the increasing use of fusion imaging in clinical practice, there is now a greater emphasis on the development of innovative SPECT or PET radiopharmaceuticals or the acquisition of more advanced SPECT or PET/CT systems. These improvements are aimed at obtaining images with a higher resolution and at a faster rate than traditional tests.

### 6.1. Gallium-68-Citrate (^68^Ga)

^68^Ga in the form of ^68^Ga-citrate is superior to ^67^Ga-citrate for the diagnosing of bone infections. ^68^Ga has a shorter physical half-life (68 min) than ^67^Ga, allowing for higher tracer doses and lower patient dosimetry. Images are obtained a few hours after radiopharmaceutical administration with better image quality than those obtained with ^67^Ga. Unfortunately, the mechanisms of ^68^Ga uptake in inflammation, trauma, malignancies, and infection are the same nonspecific mechanisms responsible for ^67^Ga uptake [2,32]. The role of ^68^Ga-citrate imaging in MSK infections has not been well studied yet, and only limited data are available on this topic. In a study by Nanni et al. [113], ^68^Ga-citrate was found to have a sensitivity of 100% and specificity of 76% in spinal OM. In a study conducted by Tseng et al. [114], 34 patients with suspected prosthetic hip/knee joint infections underwent ^68^Ga-citrate PET/CT and sequential 18F-FDG PET/CT imaging. The study found that ^68^Ga-citrate had a higher specificity and comparable sensitivity to 18F-FDG PET/CT for detecting infections. Additionally, it successfully distinguished between infectious conditions and sterile inflammation [114].

### 6.2. Technetium-Labelled Interleukin-8 (IL-8)

Interleukin-8 (IL-8) is a neutrophil-related cytokine involved in chemotaxis at the beginning of bacterial infection and innate immune response [115]. In rabbit models of OM, IL-8 labeled with ^99m^Tc was capable of discovering OM lesions [116]. The first clinical assessment of ^99m^Tc-IL-8 scintigraphy indicated favorable tolerance to tracer injection, enabling the identification of diverse infections in patients at the 4-h mark post-injection. Significantly, the tracer exhibited accumulation in cases of OM, but remained absent in non-infectious conditions, such as joint prostheses with aseptic loosening [117].

### 6.3. Sodium Fluoride (18F-NaF)

The use of sodium fluoride (18F-NaF) combined with PET/CT has recently been re-introduced. The uptake of 18F-NaF is a function of osseous blood flow and bone remodeling, which makes this scan extremely sensitive for studying bone remodeling secondary to infection/inflammation. The primary advantage of 18F-NaF is that it is not affected by marrow activity, as the radiotracer primarily represents cortical osteoblastic function. Therefore, 18F-NaF PET/CT could be an alternative to triphasic ^99m^Tc due to its increased spatial resolution and sensitivity and improved target-to-background ratio [118]. Disadvantages include a higher cost, slightly higher radiation dose, and potentially higher false-positive rate due to increased uptake at sites of degenerative changes [80,119]. A study of 23 patients with suspected post-surgical bone infection who underwent dual-phase F-18-NaF bone PET/CT showed a sensitivity, specificity, and accuracy of 92.9%, 100.0%, and 95.7%, respectively [120]. A systematic review studied dynamic or multi-sequential 18F-NaF PET in diagnosing and distinguishing between septic and aseptic loosening in hip prosthesis. They reported a diagnostic sensitivity of 97.04% and specificity of 88.11% for identifying PJI with 18F-NaF PET [121]. In this line, an ongoing clinical trial (NCT04842071) is exploring the safety and efficacy of 18F-NaF with PET/CT to replace triphasic 99mTc.

### 6.4. Radiolabeled Antibiotics

Some authors have created new tracers based on antibiotic molecules to increase the specificity in diagnosing bone infections, most of which were labeled with ^99m^-Tc for SPECT imaging. Some examples of these tracers are ^99m^Tc-ciprofloxacin, ^99m^Tc-enrofloxacin, ^99m^Tc-norfloxacin, ^99m^Tc-cephazolin, ^99m^Tc-ceftizoxime, ^99m^Tc-ertapenem, ^99m^Tc -isoniazid or ^99m^Tc-metronidazole; the major difficulty in antibiotic infection therapy and ^99m^Tc-antibiotics imaging is the appearance of resistance [122].

The most extensively studied radiolabeled antibiotic for MSK infections is ^99m^Tc-ciprofloxacin, which has demonstrated high sensitivity and specificity for the diagnosis of MSK infections in the reported studies [123]. However, caution should be exercised in instances of excessive neoformation of bone, aseptic osteoarticular diseases, and primary bone tumors, as these scenarios could yield false-positive outcomes [124]. In cases of suspected PJI, certain studies have reported the superiority of ^99m^Tc-ciprofloxacin when compared to bone/gallium imaging and labeled leukocyte BS [125,126]. It may even prove useful for monitoring the response to antibiotics [127]. Although these tracers have presented promising concepts and favorable initial outcomes, their current state of development renders them premature for prospective routine clinical application in the short term.

One limitation associated with labeling antibiotics using radiometals, like 99m-Tc, is the potential to impact the probe’s ability to enter the bacterium or interfere with its binding to the intracellular target. To circumvent the use of radiometals, individuals may consider employing PET-compatible “organic” radionuclides, like 13N or 11C, or 18Fs, such as 18F-linezolid or 11C-isoniazid/rifampicin/pyrazinamide, that have been tested in animal models [128,129]. These options are expected to have a minimal impact on the molecular structure of an antibiotic owing to their relatively small atomic radius.

Interestingly, [^11^C]-trimethoprim ([^11^C] TMP) has also been tested in human studies with PET/TC. In Lee et al.’s study, they used [^11^C]-TMP PET/CT in patients infected with both TMP-sensitive and TMP-resistant organisms and demonstrated focal radiotracer uptake in areas of infectious lesions [130]. Another promising PET tracer based on antibiotics is 18F-fluoropropil-trimetoprim ([18F] F-TMP), an analog bacterial dihydrofolate reductase inhibitor that has been used with PET and has showed that it could differentiate between infection, chemical inflammation, and tumors in rat models [131]. An ongoing clinical trial about ([18F] F-TMP) PET/CT is being carrying out to evaluate biodistribution and kinetics in human subjects (NCT04263792).

### 6.5. ^99^mTc-Ubiquicidin-29-41

Ubiquicidin (UBI) is a cationic, synthetic antimicrobial peptide fragment that has been labeled with ^99m^Tc (^99m^Tc-UBI 29-41) and used with SPECT/TC for the diagnosis of MSK infections. It has demonstrated satisfactory sensitivity and scan accuracy when employed with SPECT/CT [132]. A recent multicenter study compared MRI, 18F-FDG PET/CT, and 99mTc-UBI 29-41 scintigraphy for post-operative SD [133], but 99mTc-UBI 29-41 scintigraphy exhibited unacceptably low sensitivity and specificity.

### 6.6. Innovations in PET/CT Utilization

#### 6.6.1. Novel PET Tracers

Given the inherent lack of specificity associated with increased FDG uptake, growing interest has emerged in the investigation of alternative PET radiopharmaceuticals. Examples of these novel PET tracers include antimicrobial peptides (ubiquicidin labeled with Gallium-68- or ^68^Ga-NOTA-UBI) and carbohydrates (2-deoxy-2-18F-fluoroacetamido-D-glucopyranose or 18F-maltohexaose, 18F-fluoromaltose or 18F-Fluoromaltotriose), nanoparticles (2-Hydroxypropyl-β-CD), etc. [115,128,134]. Other promising PET tracers are based on radiolabeled antibodies, for example against Staphylococcus aureus surface molecule lipoteichoic acid labeled with Zirkonium-89 (89Zr-SAC55). This PET tracer has been investigated in a mouse model of PJI [135]. Twenty-four hours after injection, 89Zr-SAC55 exhibited notably elevated uptake at sites of S. aureus-infected prostheses compared to sterile prosthesis sites. Other potential tracers are radiolabeled molecules involved in bacteria-specific metabolism, such as p-aminobenzoic acid (PABA) labeled with Fluorine-18 [136], which targets Gram-positives, Gram-negatives, and Mycobacterium tuberculosis, and fluorodeoxysorbitol labeled with Fluorine-18, which specifically targets Enterobacteriaceae [137]. The majority of these innovative tracers have primarily been employed in vitro, animal models, or limited human-based research environments.

Gallium-68-labeled fibroblast-activation protein inhibitor (68Ga-FAPI-04) has been developed as tracer specific for fibroblast-activation protein (FAP), which is overexpressed in activated fibroblasts in various type of cancers and autoimmune diseases, and which may be a potential PET tracer in MSK infections but no studies in humans up to now have explored it in these suspected cases. Wang et al. [138] investigated 18F-FDG and 68Ga-FAPI in PJI and aseptic loosening in rabbit models. 68Ga-FAPI showed greater sensitivity and accuracy than 18F-FDG in detecting disease, but the SUVmax of 68 Ga-FAPI could not differentiate between loosening and infection. Two recent studies explored 68Ga-FAPI-04 PET/CT in rheumatic diseases, specifically in SAPHO syndrome and rheumatoid arthritis [139,140], showing encouraging results in detecting synovitis and bone lesions, and there is an ongoing clinical trial in the diagnosis of spondyloarthritis (NCT05999643). Qin et al. [141] investigated 68Ga-FAPI-04 using PET-MRI to differentiate between benign and malignant bone lesions. However, both types of lesions exhibited uptake of 68Ga-FAPI-04, although the mean SUVmax of bone metastases was significantly higher.

I-124-Fialuridine ([124I] FIAU) is a specific substrate for bacterial thymidine kinase, which is incorporated into bacteria and is expected to be specific to the infectious process. [124I] FIAU with PET/CT (FIAU-PET/CT) has been proposed [142] as an option for looking for infection in traumatized bones and prosthetic joints, but the results have been conflicting [143,144].

D-methyl-11C-methionine (exogenous d-amino acids in bacteria) is another new promising bacteria-specific tracer that has shown in a murine infection model that it can distinguish active bacterial infection from sterile inflammation and is sensitive to both Gram-positive and Gram-negative bacteria [145]. Neumann et al. [146] demonstrated a swift buildup of D-methyl-11C-methionine in mice infected with E. coli and S. aureus, with no accumulation observed in the control group with sterile inflammation. Polvoy et al. [147] has examined D-methyl-11C-methionine with PET/MRI in patients with suspected PJI and observed increased uptake in suspicious regions, demonstrating a favorable safety profile.

#### 6.6.2. New PET/CT Applications

The distribution of radiopharmaceuticals is a dynamic process that significantly varies among tumors, normal organs, and among patients. The time intervals for radiotracer uptake in clinical protocols are somewhat arbitrary, and delaying the initiation of imaging might enhance the contrast between the organs of interest and surrounding structures. Alternative protocols incorporating dynamic acquisition of temporal images enable a more comprehensive measurement of tracer kinetics. Dynamic whole-body (DWB) multiparametric PET imaging is a multi-pass imaging strategy that may significantly enhance the existing PET technique, offering an additional dimension of kinetic information. DWB images can play a crucial role in emerging imaging applications that conduct more comprehensive assessments of the temporal dynamics of radiotracer uptake. This could be particularly significant for enhancing both diagnosis and the monitoring of therapy response [148]. Furthermore, one of the capabilities of this technique is that it can reduce body motion artifacts, thus improving the image quality [149]. Unfortunately, the integration of dynamic PET into routine clinical imaging has been limited, and there is scarce evidence regarding its application in MSK infections.

Gordon et al. [150] studied dynamic ^11^C-rifampin PET in rats with confirmed S. aureus PJI or without PJI and demonstrated a bone/plasma area under the concentration-time curve ratio of 0.14, exposures lower than previously thought. The administration of a high dose of rifampin in mice resulted in elevated bone concentrations, leading to increased efficacy in bactericidal activity. Another study by Brown et al. [151] was performed using an experimental rabbit model of post-surgical OM with dynamic 18F-FDG PET imaging at 7 and 14 days after surgery. Imaging was carried out with continuous data acquisition for a 90-min period after the administration of the tracer. At 14 days, the best discrimination of infection was obtained using data acquired ∼85 min after the administration of FDG. However, at 7 days post-surgery, a more discriminative method could not be determined.

Stecker et al. [152] investigated dynamic 18F-FDG-PET/CT in nine patients with suspected chronic OM following lower extremity fractures. Ten dynamic FDG-PET/CT examinations were performed in list mode over 5 min starting with radiopharmaceutical injection, and eight consecutive time intervals (frames), namely four 15 s and then four 60 s, were reconstructed. From the 31–45 s frame onward, the affected bone area exhibited significantly higher edSUVmax and edSUVmean values compared to the healthy contralateral region. The same group subsequently confirmed these results by expanding the number of tests performed with 12 dynamic [18] F-NaF PET/CT tests in 11 patients [153].

Another intriguing advancement in PET/CT scans involves the introduction of total body PET/CT systems equipped with a detector tube measuring up to 200 cm in length, in contrast to the traditional 20 cm tube. This innovation holds the promise of significantly reducing the overall scanning time to less than a minute, enhancing image resolution, and reducing the necessary FDG dosage. This, in turn, may enable the detection of smaller FDG avid lesions and improve the differentiation between physiologic and pathological FDG uptake [154].

## 7. Machine Learning Tools: A New Era for Nuclear Medicine?

In recent years, the appearance of artificial intelligence or deep-learning (DL) methods has rapidly expanded as a powerful and promising work tool for assisting physicians in many fields of radiological imaging for different targets. DL is a branch of machine learning whose purpose is to mimic human neuronal interactions to create complex chains of interactions. DL algorithms have the potential to be leveraged for the development of clinical decision support systems that can comprehensively analyze various aspects of a patient’s medical record. In the context of MSK infections, machine learning techniques offer numerous applications, assisting physicians in more efficiently interpreting pathological images, expediting diagnoses, and streamlining infection monitoring.

Several works have investigated the potential of DL-based algorithms for analyzing bone scintigraphy scans as clinical decision-support tools. These algorithms aim to reduce the time required by nuclear physicians and to enhance image resolution. In the field of oncology, convolutional neural networks (CNNs) have been employed to automatically detect bone metastatic lesions in patients with cancer using whole-body BS images with ^99m^Tc methylene diphosphonate. Several studies [155,156] have demonstrated high specificity and sensitivity comparable to that of experienced physicians [157], without significant differences. CNN models have also been developed to enhance low-quality SPECT/CT myocardial images by reducing noise, decreasing acquisition time, and reducing the number of projections [158,159].

In a recent study, Pan et al. [160] collected anonymous scintigraphy ^99m^Tc-MDP with SPECT/CT bone image pairs, enhanced the ultra-fast acquired image using a deep neural network (U2-Net model), and quantitatively evaluated the quality of the augmented images. The U2-Net model showed that an undiagnosable SPECT image could be enhanced by the DL method to be comparable to the standard SPECT in terms of visual effect, and achieved the best performance compared to the other four advanced DL methods. In addition, the proposed method achieved an acquisition time reduction of seven times. Another study conducted by Park et al. [161] applied a new approach (pix2pix method), which incorporated a generative adversarial network (GAN) to improve blood pool images in three-phase BS when delayed images could not be obtained. The method predicted increased bone uptake matching the location of the hyperemia in the blood pool images. While the model exhibited strong performance in patients with inflammatory arthritis and complex regional pain syndrome, its sensitivity was notably lower in cases of OM, cellulitis, or recent bone injuries.

Several studies have investigated the application of a multi-task CNN algorithm for automated lesion detection and anatomical localization in whole-body bone scintigraphy, demonstrating high precision [162,163,164]. The performance of the AI models in detecting bone lesions on scintigraphy was comparable to that of human observers, with much lower time consumption [162,163].

In a retrospective study of patients with a previous diagnosis of SD, Shin et al. [165] developed a CNN model based on 18F-FDG-PET (SUVmax parameter) and blood inflammatory markers (CRP and erythrocyte sedimentation rate (ESR)) to predict SD remission. They concluded that models using SUVmax showed better performance and that the best accuracy results were obtained using all attributes (ESR, CRP, and SUVmax). Another investigation by Mukaihata et al. has been performed on DL-based algorithms based on MRI images that successfully distinguished between pyogenic spondylitis and spinal Modic changes using a CNN that interpreted MRI data [166]. These authors showed in their study an accuracy, sensitivity, and specificity of CNT greater than 80% in detecting spinal OM vs. Modic changes, with an accuracy and sensitivity equal and even superior to clinicians, surgeons, and radiologists in some MRI sequences [166]. In another study, Kim et al. [167] reported successful results with the performance of CNN in differentiating between tuberculous and pyogenic SD on MRI, and the performance was comparable to that of three skilled radiologists. Mao et al. [168] evaluated the imaging manifestations (X-ray, CT, and MRI scans) of post-operative bone and joint infections using the CNN algorithm. The CNN algorithm had a higher image quality with 90.6% sensitivity, which was higher than that of professional doctors and biomarker algorithms.

While the use of DL for interpreting NM tests yields promising initial outcomes for potential clinical applications, numerous questions and challenges remain to be addressed. Many of these studies primarily indicate the presence or absence of pathology and its anatomical location, but often do not delve into the extent of variability, differentiation from other conditions, or diagnostic uncertainties. DL models have the potential to make errors, which could lead to mistakes and harm patients receiving treatment based on the model’s predictions. DL models under development are typically trained for singular tasks, whereas patients encountered in clinical practice often present with a multitude of etiologies and conditions that necessitate intricate concurrent assessments. This could potentially be supported by upcoming technological progress, which might involve the creation of more advanced deep neural networks with large image datasets and improved graphic processing units for training machine-learning models.

To date, no studies in the existing literature have specifically investigated the use of machine learning-based interpretation of NM tests for diagnosing MSK infections, along with a comparison with conventional human-based interpretation or other imaging techniques. Therefore, there is still a significant amount of learning work in this burgeoning new field to translate current DL methods into clinical practice. Prospective and multicenter studies are also essential, encompassing MSK disorders and employing diverse imaging tests. These studies are necessary to assess the ability of these systems to evaluate pathology compared to human assessments.

## 8. Conclusions

Radionuclide imaging stands as a pivotal component in diagnosing and managing patients with suspected MSK infections, particularly when integrated with morphological imaging techniques. The advent of hybrid methods, such as SPECT/CT alongside three-phase bone scan, ^67^Ga BS, or labeled leukocyte scanning, along with 18F-FDG PET/CT, is reshaping the landscape of NM in MSK infections. Notably, 18F-FDG PET/CT yields results comparable to, and at times superior to, MRI in specific clinical scenarios, while also providing added value in monitoring treatment responses.

Nevertheless, distinguishing between inflammatory and infectious lesions using NM scans remains challenging, especially in cases involving traumatized bone or implanted metallic hardware. Looking ahead, the future holds promising prospects, including the advancement of novel radiopharmaceuticals, innovations in imaging technology, and the integration of machine learning tools. These advancements are poised to standardize and enhance the utilization of NM tests, thereby optimizing the diagnostic process and facilitating the comprehensive follow-up of patients with MSK infections.

## Data Availability

The images presented in this review originate from the nuclear medicine department, Hospital Universitario de la Princesa in Madrid, Spain. Further inquiries can be directed to the corresponding author. No other new data were created or analyzed in this study.
